# PICT-1 triggers a pro-death autophagy through inhibiting rRNA transcription and AKT/mTOR/p70S6K signaling pathway

**DOI:** 10.18632/oncotarget.12288

**Published:** 2016-09-27

**Authors:** Hongbo Chen, Yanhong Duo, Bo Hu, Zhiwei Wang, Fang Zhang, Hsiangi Tsai, Jianping Zhang, Lanzhen Zhou, Lijun Wang, Xinyu Wang, Laiqiang Huang

**Affiliations:** ^1^ The Shenzhen Key Lab of Gene and Antibody Therapy, Center for Biotechnology & Biomedicine, Division of Life and Health Sciences, Graduate School at Shenzhen, Tsinghua University, Shenzhen 518055, China; ^2^ School of Life Sciences, Tsinghua University, Beijing 100084, China; ^3^ Key Laboratory of Plant Cell Activities and Stress Adaptation, Ministry of Education, School of Life Sciences, Lanzhou University, Lanzhou 730000, China; ^4^ Department of Laboratory Medicine, The Third Affiliated Hospital of Sun Yat-sen University, Guangzhou 510630, China; ^5^ Department of Laboratory Medicine, The Fourth Affiliated Hospital of Guangzhou Medical University, Guangzhou 511447, China; ^6^ Department of Quality Inspection, Shenzhen Weiguang Biological Products Co., Ltd, Shenzhen 518107, China

**Keywords:** PICT-1, nucleolus, autophagy, rRNA transcription, p53

## Abstract

PICT-1 was originally identified as a tumor suppressor. Here, we found that PICT-1 overexpression triggered pro-death autophagy without nucleolar disruption or p53 accumulation in U251 and MCF7 cells. Truncated PICT-1 fragments 181-346 and 1-346, which partly or totally lack nucleolar localization, showed weaker autophagy-inducing effects than full-length PICT-1 and a well-defined nucleolar mutant (181-479). Furthermore, PICT-1 partly localizes to the nucleolar fibrillar center (FC) and directly binds to ribosomal DNA (rDNA) gene loci, where it interacts with upstream binding factor (UBF). Overexpression of PICT-1 or the 181-479 mutant, but not the 1-346 or 181-346 mutants, markedly inhibited the phosphorylation of UBF and the recruitment of rRNA polymerase I (Pol I) to the rDNA promoter in response to serum stimulation, thereby suppressing rRNA transcription, suggesting that rRNA transcription inhibition might be an important contributor to PICT-1-induced autophagy. This is supported by the finding that CX-5461, a specific Pol I inhibitor, also induced autophagy. In addition, both CX-5461 and PICT-1, but not the 1-346 or 181-346 mutants, significantly suppressed the activation of the Akt/mTOR/p70S6K signaling pathway. Our data show that PICT-1 triggers pro-death autophagy through inhibition of rRNA transcription and the inactivation of AKT/mTOR/p70S6K pathway, independent of nucleolar disruption and p53 activation.

## INTRODUCTION

The human “protein interacting with carboxyl terminus 1” (PICT-1), also known as human glioma tumor suppressor candidate region 2 gene product (GLTSCR2), was originally identified as a 60 kDa (p60) interacting partner of two viral proteins, ICP0 and ICP22 [1]. PICT-1 is located in the 1.4 Mb putative tumor suppressor locus of human chromosome 19q, a genetic region which is frequently altered in human tumors, particularly gliomas [2]. PICT-1 is considered to be a candidate tumor suppressor gene, as its diminished expression or loss is correlated with the highly malignant progression of several cancers [3,4]. In support of this hypothesis, research has shown that knockdown of PICT-1 promoted anchorage-independent tumor cell growth and decreased susceptibility to apoptotic cell death in response to apoptosis-inducing stimuli, whereas overexpression of PICT-1 significantly inhibited anchorage-independent tumor cell growth and induced mitochondria-independent cell death [5,6]. Investigation of the molecular mechanisms involved in these phenomena showed that PICT-1 can regulate the phosphorylation and thus the stability of the well-known tumor suppressor PTEN by direct interaction, indicating that the anti-cancer function of PICT-1 is mediated at least partly through its inhibition of the PI3K/AKT signaling pathway [7].

However, the roles and signaling mechanisms of PICT-1 in healthy and cancer cells are not yet fully understood. For example, previous research has shown that PTEN-mediated cell death is a caspase- and mitochondria-dependent pathway involving p53 activation, in contrast to PICT-1-induced caspase- and mitochondrial-independent cell death, which does not require p53 activation [5,6,8]. In addition, PICT-1 has recently been shown to preferentially localize to the nucleolus [9–13], suggesting that it may exert an important role in nucleolar function. The nucleolus is best known as a site of rDNA transcription and ribosome biogenesis, but accumulating evidence has demonstrated that the nucleolus also participates in a diverse array of cell functions such as stress response, the cell cycle, aging processes, cell death, and several human diseases [14–16].

Autophagy is a lysosomal degradation system that facilitates the breakdown of intracellular proteins and organelles. Autophagy is traditionally considered to be a protective mechanism, important for the removal of damaged proteins and organelles and conferring stress tolerance and enhancing cell viability under adverse conditions [17]. However, the functional role of autophagy in cell fate remains a contentious subject, with some research supporting the controversial view that autophagy can also serve as a pro-death pathway rather than a pro-survival mechanism under specific conditions [18,19]. Several recent studies suggest that the nucleolus has a close relationship to autophagic processes. Firstly, a number of cell stressors have been found to induce both nucleolar dysfunction and autophagy [20]. Secondly, autophagy-related signalling molecules can also regulate ribosomal RNA (rRNA) transcription and ribosomal biogenesis [21,22]. Thirdly, and most importantly, knockdown of TIF-IA, which is an essential initiation factor required for rRNA transcription, and POLR1A, a catalytic subunit of rRNA polymerase I (Pol I), induces both nucleolar disruption and the formation of autophagic vesicles, suggesting that these processes are closely associated [23,24]. Still, much remains to be discovered about which type of autophagy is induced by nucleolar dysfunction and how about the precise molecular mechanism.

In the present investigation, we found that overexpression of PICT-1 induces Akt/mTOR/p70S6K signaling pathway-related pro-death autophagy, without nucleolar disruption or p53 activation. Furthermore, the pro-autophagy effects of PICT-1 overexpression are implicated in its nuclear localization and its rDNA transcription inhibition function. Therefore, our evidence suggests that PICT-1 is a potent regulator of ribosome biogenesis and nucleolus-related autophagy formation.

## RESULTS

### The overexpression of PICT-1 induces autophagy formation in cancer cell lines

**Figure 1:**
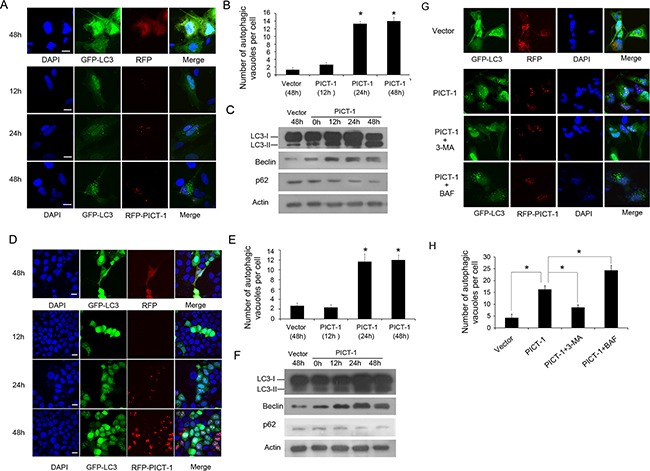
PICT-1 overexpression triggers autophagy in U251 and MCF7 cells **A.** U251 cells were co-transfected with GFP-LC-3 and dsRed-PICT-1 or control dsRed-C1 plasmids, and then observed under a confocal microscope at the indicated time points. Representative images are shown. Scale bar = 10 μm. **B.** The number of GFP-LC3-positive puncta per cell was counted and results are presented as mean ± SD (* *p* < 0.05). **C.** U251 cells were transfected with pFLAG-CMV2 or pFLAG-CMV2-PICT-1, and Western blotting was performed with antibodies against LC3, Beclin, p62 and β-actin at the indicated time points. **D.** and **E.** MCF7 cells were treated as in (A), and GFP-LC3-positive puncta were counted (mean ± SD, * *p* < 0.05). Scale bar = 10 μm. **F.** MCF7 cells were transfected with pFLAG-CMV2 or pFLAG-CMV2-PICT-1, and Western blotting was performed with antibodies against LC3, Beclin, p62 and β-actin at the indicated time points. **G.** U251 cells transfected with dsRed-PICT-1 or control dsRed-C1 plasmid were treated with or without 3-MA or BAF and then observed with confocal microscopy at 48h post-transfection. Scale bar = 10 μm. **H.** The number of GFP-LC3-positive puncta per cell was counted and the results are presented as mean ± SD (* *p* < 0.05).

Previous research has shown that PICT-1 protein is localized to the nucleolus. However, its nucleolar function and molecular mechanisms of action are still not fully understood. Autophagy is classically considered as a “self-eating” process in which a portion of the cytoplasm is sequestered within autophagosomes. The autophagosomes are then delivered to the lysosome, where their cargoes are broken down and the resulting amino acids are made available for reuse by the cell. Autophagy is therefore closely related to protein metabolism and various nutrient limitations [25]. The nucelolus is the site of ribosome biogenesis, and has been implicated in regulating protein metabolism. In order to investigate whether the nucleolar protein PICT-1 is involved in autophagy, human glioblastoma U251 cells were co-transfected with PICT-1 and GFP-LC3 plasmids, and GFP-LC3 localization was then examined by confocal microscopy. Previous studies reported that LC3-I can be converted to LC3-II during the autophagic process, which promotes the translocation of LC3-II into autophagic vesicles [26]. For this reason the presence of LC3-II in autophagic vesicles and the conversion of LC3-I to LC3-II can be used as markers of the induction of autophagy. As shown in Figure 1A and 1B, the number of autophagic vesicles displaying a punctate staining pattern for GFP-LC3-II was significantly increased in PICT-1-overexpressing U251 cells in a time-dependent manner compared with control cells. Additionally, the ratio of LC3-II to LC3-I was significantly increased in U251 cells transfected with the PICT-1 plasmid in a time-dependent manner (Figure 1C). The protein levels of another two autophagy-related markers Beclin 1 and p62 were also determined by Western blot. As shown in Figure 1C, PICT-1 overexpression significantly increased the level of Beclin 1, but decreased the level of p62. Similar results were observed in MCF7 breast cancer cells (Figure 1D, 1E and 1F). To further confirm these results, U251 cells overexpressing PICT-1 were treated with two autophagy inhibitors 3-methyladenine (3-MA, 10 mM) and bafilomycin (BAF, 100 nM), which are known to inhibit the initiation of autophagosome and the fusion of autophagosome with lysosome, respectively. As shown in Figure 1G and 1H, 3-MA blocked the formation of GFP-LC3 autophagic vesicles induced by PICT-1 overexpression. In contrast, BAF enhanced formation of the punctate LC3. Collectively, these data suggest that PICT-1 overexpression indeed significantly induced autophagy in cancer cells.

### The ability of PICT-1 to induce autophagy is related to its nucleolar localization

**Figure 2:**
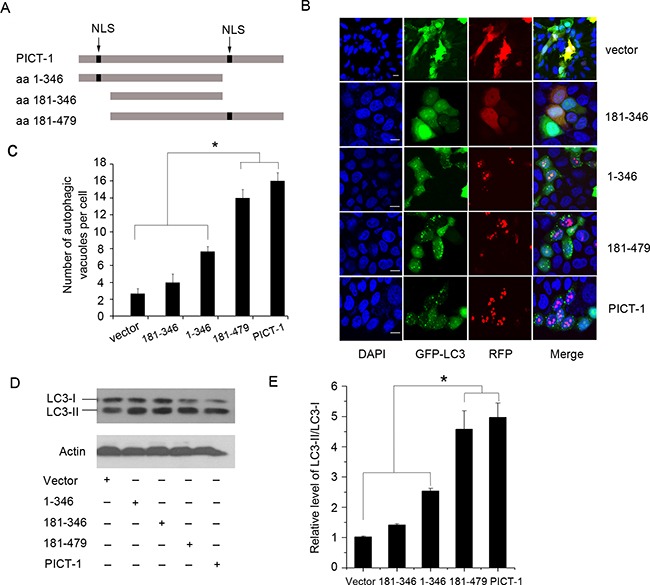
The nucleolar accumulation of PICT-1 is required for PICT-1-induced autophagy **A.** Schematic representation of PICT-1 and its truncation mutants (NLSs, the presumed nuclear localization signals). **B.** MCF7 cells were co-transfected with GFP-LC3 and dsRed-PICT-1, dsRed-PICT-1 (1-346), dsRed-PICT-1 (181-346), or dsRed-PICT-1 (181-479), and then observed under a confocal microscope. Representative images are shown. Scale bar = 10 μm. **C.** The number of GFP-LC3-positive puncta per cell was counted and results are presented as mean ± SD (* *p* < 0.05). **D.** MCF7 cells were transfected with pFLAG-CMV2-PICT-1, pFLAG-CMV2-PICT-1 (1-346), pFLAG-CMV2-PICT-1 (181-346), pFLAG-CMV2-PICT-1 (181-479), or pFLAG-CMV2 control vector, and Western blotting was performed with LC3 and β-actin antibodies 24 h post-transfection. **E.** Protein levels were quantified by scanning densitometry and the expression ratios of LC3-II/LC3-I were calculated. Data are expressed as relative fold of control plasmid treatment (* *p* < 0.05).

Previous research has identified two classical nuclear localization sequences (NLSs) and a non-classical, unique nucleolar localization sequences (NoLS) on PICT-1 [6,10,11]. Based on these findings, we constructed PICT-1 truncation mutants of amino acid (aa) 1-346 (containing the amino-terminal NLS), aa 181-346 (deleting the two NLSs), and aa 181-479 (containing the carboxyl-terminal NLS and the non-classical NoLS) (Figure 2A). In agreement with previous reports, we found that both full-length PICT-1 and the 181–479 fragment had a definite pattern of nucleolar localization in MCF7 cells. In contrast, the 181–346 mutant was dispersed throughout the cytoplasm. Although the 1-346 fragments primarily exhibited nucleolar globular expression, we also observed some diffuse distribution throughout the nucleus. As shown in Figure 2B and 2C, the number of autophagic vesicles in cells expressing full-length PICT-1 or the 181–479 fragment was significantly greater than in the cells expressing the 1-346 mutant protein. In contrast, cells expressing the 181-346 fragment had the least number of GFP-LC3-II-positive autophagic vesicles. Western blotting also showed that the ratio of LC3-II to LC3-I is significantly higher in cells with full-length PICT-1 or 181–479 overexpression than in cells overexpressing either the 1-346 or 181-346 fragments (Figure 2D and 2E). These data indicate that the ability of PICT-1 to induce autophagy depends on its localization to the nucleolus.

### PICT-1 inhibits the transcription of rDNA and proliferation of U251 cells

**Figure 3:**
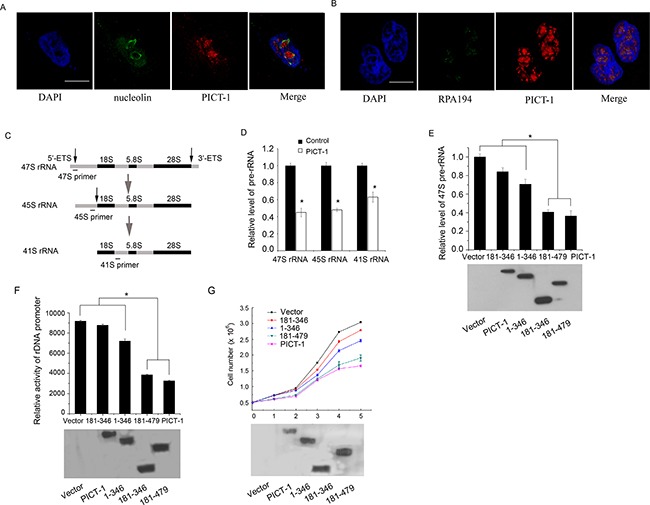
PICT-1 partly localizes to nucleolar FC region, and can regulate rRNA transcription and inhibit cell growth and proliferation **A.** and **B.** The co-localization of the endogenous PICT-1 with nucleolin (nucleolar GC marker) or RPA194 (FC marker) in MCF7 cells was investigated by immunofluorescence. Scale bar = 10 μm. **C.** Schematic representation of 47S, 45S and 41S pre-rRNA and the position of primers used for qPCR to detect nascent pre-rRNA levels. **D.** U251 cells were transfected with FLAG-PICT-1 or control plasmid (lower panel). Total RNA was extracted 48 h post-transfection, and qPCR was performed with primers shown in (C). The data from three independent experiments are presented as relative levels of vector treatment, and 47S, 45S and 41S pre-rRNA levels in the control were set to 1. **E.** U251 cells were transfected with FLAG-PICT-1 or a FLAG-PICT-1 truncation mutant plasmid (lower panel). 47S pre-rRNA was detected by qPCR at 48 h post-transfection. The pre-rRNA level in the control was set to 1 (* *p* < 0.05) (upper panel). **F.** U251 cells were co-transfected with pHrD-IRES-Luc and pFLAG-CMV2-PICT-1 or one of its mutant plasmids, and the expression level of FLAG-PICT-1 or its mutant plasmids was detected (lower panel). After 48 h, cell lysates containing comparable amounts of protein were subjected to luciferase activity analysis. Data was presented as the mean ± SD (in arbitrary units, AU) of three independent experiments (* P<0.05) (upper panel). **G.** U251 cells were transfected with FLAG-PICT-1 or a PICT-1 truncation mutant plasmid (lower panel), and cell numbers were determined every 24 h. Results are expressed as mean ± SD for three replicates (upper panel).

The nucleolus is classically considered to be the site of ribosome biogenesis, which includes rDNA transcription, nascent rRNA processing, and the assembly of ribosomal subunits. The ultrastructure of the nucleolus when visualized by electron microscopy is composed of fibrillar centers (FC), dense fibrillar components (DFC), and granular components (GC) [27,28]. Transcription of the rDNA gene repeats by Pol I occurs mainly at the border between the FC and DFC, the processing of nascent rRNA occurs largely in the DFC region, while the GC region consists of proteins that are necessary to complete the assembly of ribosomal subunits. Generally, the functions of nucleolar proteins are related to their sub-nucleolar localization. To begin to explore the role of PICT-1 in the nucleolus, the co-localization of PICT-1 with two different sub-nucleolar markers was investigated using confocal microscopy. As shown in Figure 3A, PICT-1 shows no co-localization with the GC region marker nucleolin [29], whereas PICT-1 partly co-localizes with the FC marker RPA194 (a subunit of Pol I) in MCF7 cells [30], indicating that PICT-1 might participate in the regulation of rDNA transcription (Figure 3B).

The initial transcriptional product of rDNA is known as 47S pre-rRNA. Processing of rRNA in mammalian cells includes a series of cleavages of the primary 47s transcript and results in producing several intermediate rRNAs, such as 45S, 41S and 36S rRNA, and three mature rRNAs: 18s, 28s and 5.8s (Figure 3C). Because the 5′ external transcribed spacer (5′-ETS) can be rapidly cleaved from pre-rRNA, the level of 5′-ETS can be used to assay the rate of rDNA transcription. To investigate the effect of PICT-1 on pre-rRNA transcription, U251 cells were transfected with FLAG-PICT-1 or control plasmid for 48 h, and the amount of 47S, 45S and 41S rRNA was quantified by qPCR using primers described by previous reports [31, 32]. As shown in Figure 3D, both 47S, 45S and 41S pre-rRNA levels were significantly lower in cells transfected with PICT-1 compared with cells transfected with control plasmid, which suggested that PICT-1 overexpression significantly suppresses the transcription of the rDNA gene. Furthermore, U251 cells were transfected with full-length PICT-1 or one of its three truncation mutants and the amount of 47S pre-rRNA was quantified by qPCR. As shown in Figure 3E, a marked decrease in pre-rRNA levels was observed in cells transfected with full-length PICT-1 compared with cells transfected with control plasmid, suggesting that PICT-1 overexpression significantly suppressed the transcription of the rDNA gene. The 181-479 mutant protein had an inhibitory effect on rDNA transcription similar to that of full-length PICT-1, whereas the 1-346 and 181-346 fragments more weakly inhibited rDNA transcription than full-length PICT-1 or the 181-479 mutant.

To further confirm this inhibitory effect of PICT-1 on rDNA transcription, a pHrD-IRES-Luc plasmid [33,34] containing the human rRNA promoter sequence was co-transfected into U251 cells together with PICT-1 mutant plasmids, and luciferase activity was measured 48 h after transfection. Similar to the qPCR results, full-length PICT-1 and the 181-479 mutant showed a greater inhibitory effect on rRNA promoter activity compared with either the 1-346 or 181-346 mutants (Figure 3F).

Because ribosome biogenesis is required for cell growth and proliferation [35], we next investigated the effect of PICT-1 truncation mutant overexpression on cell proliferation. As expected, both full-length PICT-1 and the 181-479 mutant exhibited a greater inhibitory effect on proliferation of U251 cells than did the 1-346 and 181-346 mutants (Figure 3G). Collectively, these data suggest that PICT-1 overexpression inhibits rDNA transcription and cell proliferation, and that this inhibitory effect is related to its nucleolar localization.

### PICT-1 can bind to rDNA gene loci and regulate Pol I transcriptional machinery activity

**Figure 4:**
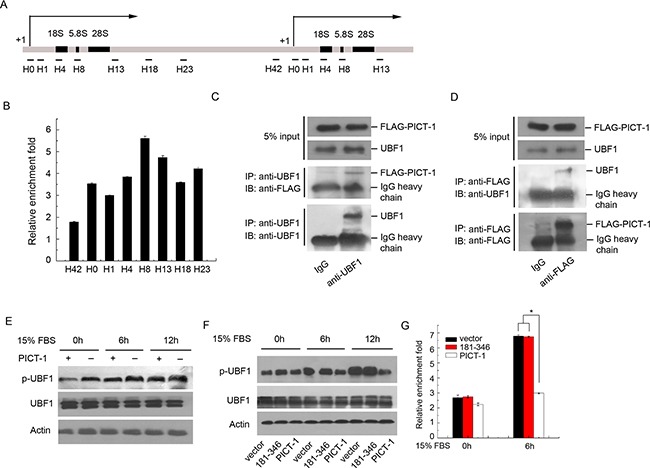
PICT-1 occupies rDNA loci and regulates UBF1 activity and the recruitment of Pol I to the rDNA promoter **A.** Schematic illustration of rDNA gene repeat and the positions of primers used for ChIP. The numbers (H0-H42) are the distances (kb) from the transcriptional start site (+1) across the rDNA loci. **B.** ChIP assay was performed with control IgG or anti-FLAG antibody and the precipitated DNA from U251 cells was analyzed by qPCR using the primers described in (A). The data from samples treated with FLAG antibody are expressed as relative enrichment fold of IgG treatment. **C.** U251 cells were transfected with pFLAG-CMV2-PICT-1 plasmid, and immunoprecipitation assays were performed using anti-UBF1 or control IgG antibody 24 h post-transfection. The co-precipitated FLAG-PICT-1 was examined by Western blotting using anti-FLAG antibody. **D.** A converse IP using anti-FLAG was performed and the co-precipitated UBF1 was examined by Western blotting using anti-UBF1 antibody. **E.** U251 cells transfected with pFLAG-CMV2-PICT-1 or control vector were serum starved for 24 h and then stimulated with 15% serum. Levels of p-UBF were detected by Western blotting at the indicated time points. **F.** U251 cells transfected with control vector, pFLAG-CMV2-PICT-1 (181-346), or pFLAG-CMV2-PICT-1 were serum starved for 24 h and then stimulated with 15% serum. Levels of p-UBF were detected by Western blotting at the indicated time points. **G.** U251 cells transfected with control vector, pFLAG-CMV2-PICT-1 (181-346), or pFLAG-CMV2-PICT-1 were serum starved for 24 h and then stimulated with 15% serum. Equal amounts of the cell lysates were used for ChIP assays with anti-RPA194 or control IgG antibody at the indicated time points. The precipitated DNA was analyzed by qPCR with the primers for H0. Data from three independent experiments are expressed as relative fold-enrichments of IgG treatment (* *p* < 0.05).

To determine whether PICT-1 directly associates with the rDNA gene, we performed ChIP analysis in U251 cells with multiple PCR primer sets targeting the rDNA loci [36]. As shown in Figure 4B, PICT-1 binding on the entire rDNA gene loci was enriched 2- to 6-fold versus IgG controls, suggesting that PICT-1 might participate in the transcriptional regulation of rDNA by directly binding to the rDNA genetic loci. Transcription of rDNA also requires the assembly of Pol I transcriptional machinery, and the activation of upstream binding factor 1 (UBF1) is a key step in the recruitment of RNA polymerase I to the rDNA loci [37]. Several rDNA-binding proteins have been shown to regulate the activity of Pol I transcriptional machinery through their interactions with these factors [16,38]. We used co-immunoprecipitation assays to determine whether PICT-1 associates with the Pol I transcriptional machinery, and found that PICT-1 specifically interacts with UBF1 (Figure 4C and 4D) but not with RPA194 (Data not shown). It has previously been reported that the phosphorylation of UBF1 at ser388 is required for its activation, and that phosphorylation of UBF1 is significantly increased in response to growth factor stimulation [39,40]. To determine whether PICT-1 can affect UBF1 activation, U251 cells transfected with PICT-1 plasmid for 24 h were starved in serum-free DMEM for another 24 h, and then stimulated with 15% serum for the indicated times. The data depicted in Figure 4E show that PICT-1 overexpression markedly suppressed UBF1 phosphorylation at ser388 in response to serum stimulation. We also found that the 181-346 mutant, which lacks both NLSs, showed no obvious inhibitory effect on serum-stimulated UBF1 phosphorylation compared with full-length PICT-1 (Figure 4F). Several studies have shown that the phosphorylation and activation of UBF1 are important for the recruitment of the Pol I complex to rDNA loci and the subsequent transcription of rRNA [41,42]. Our ChIP assay targeting the rDNA promoter region using the RPA194 antibody showed that full-length PICT-1, but not the 181-346 truncation mutant, significantly suppressed the binding of RPA194 to rDNA loci (Figure 4G). Taken together, these results suggest that PICT-1 can bind to rDNA genetic loci and interact with UBF1. PICT-1 overexpression inhibits the activation of UBF1 and the recruitment of Pol I to rDNA loci, thereby impacting rDNA transcription.

### PICT-1-induced autophagy involves rRNA transcription inhibition and the AKT/mTOR/p70S6K pathway

**Figure 5:**
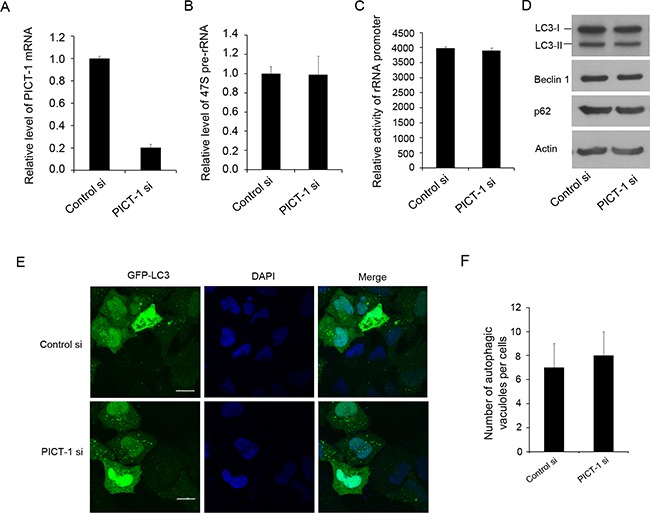
The knockdown of PICT-1 does not significantly affect the pre-rRNA transcription and induce autophagic formation **A.** U251 cells were transfected with control or PICT-1-specific siRNA for 48 h, total RNA was extracted and qPCR was performed for PICT-1 mRNA and **B.** 47S pre-rRNA. The data are presented as relative levels of control siRNA, and PICT-1 mRNA and pre-rRNA levels in the control were set to 1. **C.** U251 cells were co-transfected with pHrD-IRES-Luc and control or PICT-1-specific siRNA. After 48 h, cell lysates containing comparable amounts of proteins were subjected to luciferase activity analysis. Data are presented as mean ± SD (in arbitrary units, AU) of three independent experiments. **D.** U251 cells were transfected with control or PICT-1-specific siRNA for 48 h, and Western blotting was performed with antibodies against PICT-1, LC3, Beclin, p62 or β-actin. **E.** U251 cells were co-transfected with GFP-LC3 plasmid and control or PICT-1-specific siRNA for 48 h, and imaged under a confocal microscope (scale bar = 10 μm). **F.** The number of GFP-LC3-positive puncta per cell was counted.

**Figure 6:**
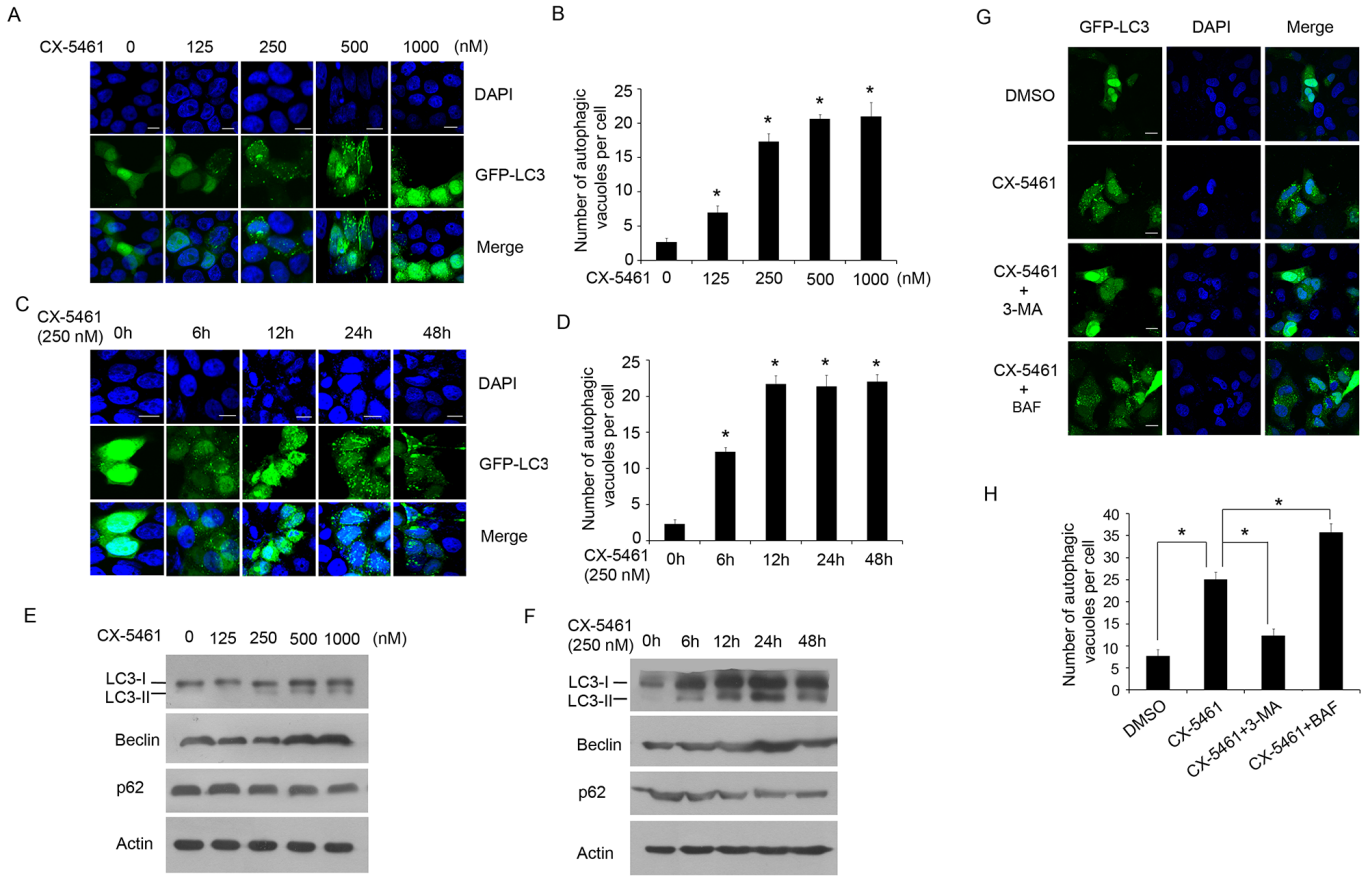
CX-5461, a specific inhibitor of Pol I, can also induce autophagy **A.** U251 cells were transfected with GFP-LC-3 plasmid for 24 h, and then treated with CX-5461 at the indicated concentrations for another 24 h. The cells were imaged under a confocal microscope (scale bar = 10 μm), and **B.** the number of GFP-LC3-positive puncta per cell was counted (* *p* < 0.05). **C.** U251 cells were transfected with GFP-LC-3 plasmid for 24 h, and then treated with 250 nM of CX-5461. The cells were imaged under a confocal microscope at the indicated time points post-treatment (scale bar = 10 μm), and **D.** the number of GFP-LC3-positive puncta per cell was counted (* *p* < 0.05). **E.** and **F.** U251 cells were treated as in (A) or (C), and Western blotting was performed with LC3, Beclin, p62 and β-actin antibodies. **G.** U251 cells transfected with GFP-LC-3 plasmid for 24 h, and then treated with 250 nM of CX-5461 in the presence or absence of 3-MA or BAF. The cells were imaged under a confocal microscope (scale bar = 10 μm). **H.** The number of GFP-LC3-positive puncta per cell was counted (* *p* < 0.05).

As described above, the overexpression of PICT-1 can inhibit rRNA transcription and induce autophagy in U251 and MCF7 cells. Here, we also investigated the impact of PICT-1 knockdown (Figure 5A) on rRNA biogenesis and autophagy. As shown in Figure 5B and C, we did not observe significant changes in pre-rRNA level (Figure 5B) and activity of rDNA promoter (Figure 5C) induced by PICT-1 knockdown. Similarly, we did not observe significant changes in expression levels of autophagy-related markers (Figure 5D) as well as induction of autophagy in U251 cells (Figure 5E and 5F). These results further comfirm that inhibition of rRNA transcription might be a key factor for PICT-1 overexpression-induced autophagy. However, the mechanistic relationship between inhibition of rRNA transcription and autophagy is still elusive. To test our hypothesis, CX-5461 was used to selectively inhibit rRNA transcription and autophagy was subsequently investigated. CX-5461 has been found to selectively block rRNA synthesis by inhibiting nucleolar Pol I polymerase, but it does not inhibit mRNA synthesis (Pol II), DNA replication, or protein synthesis [43,44]. To test whether CX-5461 induces autophagy, U251 cells transfected with GFP-LC3 were treated with CX-5461 and autophagic vesicles were quantified. As we predicted, the number of autophagic vesicles was significantly increased in CX-5461-treated cells when compared with untreated cells in both a dose- and time-dependent manner (Figure 6A, 6B, 6C and 6D). Consistently, CX-5461 treatment induced a dose- and time-dependent increase in LC3-II and Beclin 1 levels, but a decrease in p62 level in U251 cells (Figure 6E and 6F). U251 cells were also treated with CX-5461 (250 nM) in the presence or absence of 3-MA or BAF, and autophagy was analyzed by confocal microscopy. As shown in Figure 6G and 6H, 3-MA inhibited the formation of GFP-LC3 autophagic vesicles induced by CX-5461, while BAF increased the formation of punctate LC3. These results suggest that CX-5461 can induce autophagy in cancer cells.

**Figure 7:**
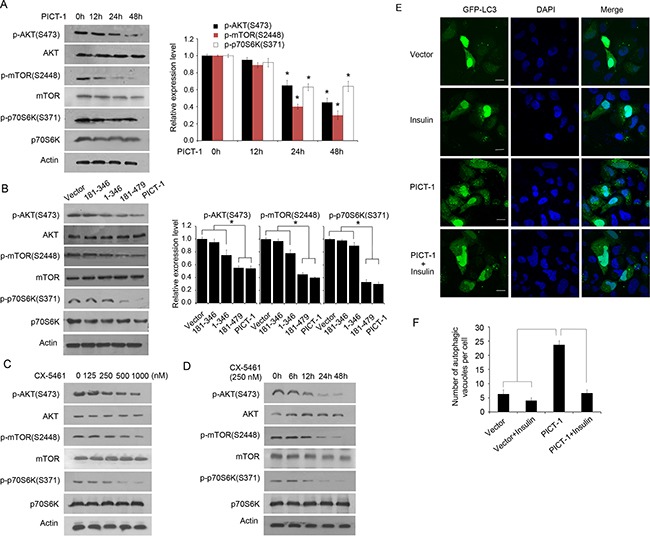
PICT-1- and CX-5461-induced autophagy involve the AKT/mTOR/p70S6K pathway **A.** U251 cells were transfected with pFLAG-CMV2-PICT-1, and Western blotting was performed with the indicated antibodies at 0 h, 12 h, 24h and 48 h post-transfection. *Column:* Protein levels were quantified by scanning densitometry and the relative expression levels of p-AKT (S473)/AKT, p-mTOR (S2448)/mTOR, and p-p70S6K (S371)/Actin were calculated. The data from 0 h was set to 1 (* *p* < 0.05). **B.** U251 cells were transfected with control vector, PICT-1, or a PICT-1 mutant plasmid, and Western blotting was performed with the indicated antibodies 48 h post-transfection. *Column:* Protein levels were quantified by scanning densitometry and the relative expression levels of p-AKT (S473)/AKT, p-mTOR (S2448)/mTOR, and p-p70S6K (S371)/Actin were calculated. The data from control vector treatment was set to 1 (* *p* < 0.05). **C.** U251 cells were treated with CX-5461 at the indicated concentrations for 24 h, and Western blotting was performed with the indicated antibodies. **D.** U251 cells were treated with 250 nM of CX-5461, and Western blotting was performed at the indicated time points. **E.** U251 cells were transfected with pFLAG-CMV2 vector or pFLAG-CMV2-PICT-1 in presence or absence of insulin (1 nM), and autophagic vesicles were observed with confocal microscopy at 48 h after transfection. **F.** The number of GFP-LC3-positive puncta per cell was counted (* *p* < 0.05).

The AKT/mammalian target of rapamycin (mTOR)/p70 ribosomal protein S6 kinase (p70S6K) signaling pathway is known to regulate autophagy [18,45]. To investigate whether CX-5461- or PICT-1-induced autophagy involves the Akt/mTOR/p70S6K signaling pathway, we used Western blots to quantify the phosphorylation levels of the associated proteins. As shown in Figure 7A, PICT-1 overexpression significantly inhibited the phosphorylation of Akt, mTOR, and p70S6K in a time-dependent manner. In addition, overexpression of the 181-479 PICT-1 truncation mutant and full-length PICT-1 significantly reduced the levels of phosphorylated Akt, mTOR, and p70S6K compared with the 1-346 and 181-346 mutants (Figure 7B), indicating that the nucleolar localization and rRNA transcription-inhibiting ability of PICT-1 are important for the inhibition of Akt/mTOR/p70S6K signaling. Similarly, CX-5461 treatment also resulted in decreased phosphorylation of Akt, mTOR, and p70S6K compared with untreated U251 cells in both a dose- and time-dependent manner (Figure 7C and 7D). Insulin can activate the Akt/mTOR pathway, therefore we further investigated the effect of insulin on the autophagy formation induced by PICT-1 overexpression. As shown in Figure 7E and 7F, insulin treatment significantly attenuated the PICT-1 overexpression-induced autophagy in U251 cells. Taken together, these data suggest that PICT-1-induced autophagy involves inhibition of both rRNA transcription and the mTOR/P70S6K signaling pathway.

### The autophagy induced by PICT-1 is a pro-death process without nucleolar disruption and p53 activation

**Figure 8:**
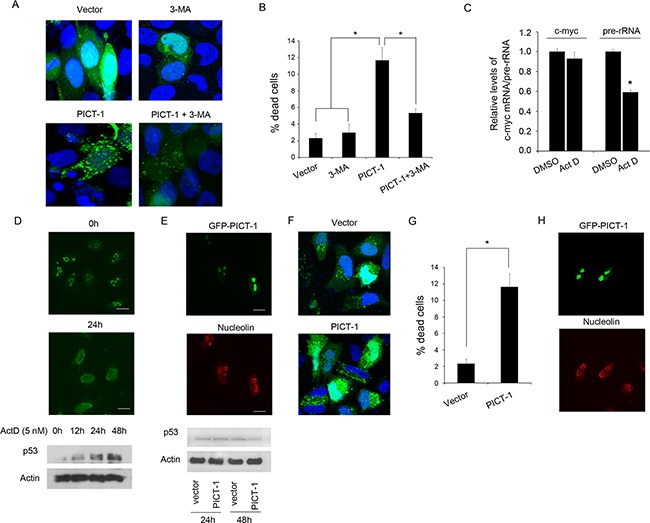
The autophagy induced by PICT-1 is a pro-death process without p53 activation **A.** U251 cells co-transfected with GFP-LC3 and pFLAG-CMV2-PICT-1 or control vector plasmid were treated with 3-MA or vehicle. The cells were observed under a confocal microscope (green fluorescence represents GFP-LC3, while blue fluorescence represents DAPI nuclear staining; scale bar = 10 μm), and **B.** dead cells were counted 48 h after transfection. Data are presented as the mean ± SD for three independent experiments (* *p* < 0.05). **C.** U251 cells were treated with or without Act D (5 nM), and the expression levels of pre-rRNA and c-myc mRNA were investigated by qPCR at 24 h post-treatment. **D.** U251 cells were treated with or without of 5 nM of Act D. *Upper images*: The cells were stained with anti-nucleolin (green) and examined by confocal microscopy at 0 h and 24 h post-treatment. *Lower lanes:* p53 levels were quantified by Western blotting at the indicated time points. **E.** U251 cells were transfected with pGFPc1-PICT-1. The localization of GFP-PICT-1 (green) and nucleolin (red) was observed by confocal microscopy at 48 h post-transfection (upper images) and p53 levels were detected by Western blotting at 24 h and 48 h post-transfection (lower lanes). **F.** H1299 cells co-transfected with GFP-LC3 and pFLAG-CMV2-PICT-1 or control vector plasmid for 48 h and observed under a confocal microscope (green fluorescence represents GFP-LC3, and blue fluorescence represents DAPI nuclear staining; scale bar = 10 μm), and **G.** dead cells were counted 48 h after transfection. Data are presented as the mean ± SD for three independent experiments (* *p* < 0.05). **H.** H1299 cells were transfected with pGFPc1-PICT-1. The localization of GFP-PICT-1 (green) and nucleolin (red) was observed by confocal microscopy at 48 h post-transfection.

As previously reported, autophagy seems to have different roles when initiated by different cellular stresses, either protecting cells or promoting cell death [17–19]. However, the functions of autophagy induced by PICT-1 overexpression in cancer cells are still unclear. To identify which type of autophagy is induced by PICT-1 overexpression, we investigated the effect of the specific inhibitor of autophagy 3-methyl adenine (3-MA) on PICT-1-induced cell death in U251 cells. As shown in Figure 8A, 3-MA treatment markedly attenuated the accumulation of GFP-LC3-II-positive autophagic vesicles in PICT-1 overexpressing cells, indicating that 3-MA inhibited the progress of autophagy by blocking autophagosome formation in the early stages of autophagy. Accordingly, nuclear staining by DAPI showed that cell death rates 48 h after PICT-1 plasmid transfection were significantly attenuated by 3-MA treatment (Figure 8B). These results suggest that the autophagy induced by PICT-1 overexpression is a growth-inhibiting and pro-death process.

Several investigations have reported that the nucleolus can serve as a stress sensor, and that its disruption can activate p53-mediated cell death when subjected to DNA damage or Pol I transcription inhibitors [46]. Moreover, p53 activation has been reported to play a role in the induction of autophagy [23]. To test whether p53 is essential for PICT-1 induced cell death and autophagy, nucleolar integrity and p53 levels were assayed in U251 cells following either actinomycin D (ActD) treatment or PICT-1 overexpression. Previous studies showed that low-dose Act D can specifically inhibits RNA polymerase I but not RNA polymerase II [24]. Here, the 47S pre-rRNA and c-myc mRNA were served as Pol I transcript and Pol II transcript, respectively. Both 47S pre-rRNA and c-Myc mRNA are short-lived RNA transcripts (half-lives 20~30 minutes) and have been used for markers to evaluate the Pol I- and Pol II-driven efficiency. In agreement with previous reports [24], treatment with low-dose Act D (5 nM) specifically inhibited RNA polymerase I but not RNA polymerase II (Figure 8C) caused p53 accumulation (Figure 8D, lower panel) concomitant with nucleolar disruption, as shown by translocation of the nucleolar marker protein nucleolin from nucleolus to nucleoplasm (Figure 8D, upper panel). However, p53 levels showed no significant increase at 24 h and 48 h after pFLAG-CMrV2-PICT-1 transfection compared with control plasmid transfection (Figure 8E, lower panel). In addition, confocal microscopy showed that PICT-1 overexpression did not obviously affect nucleolar structure or integrity (Figure 8E, upper panel). These results indicate that PICT-1-induced autophagy is a pro-death form of autophagy, which does not involve nucleolar disruption and p53 activation. Furthermore, we performed the experiments in p53-null H1299 lung cancer cell line. As shown in Figure 8F, PICT-1 overexpression significantly increased the accumulation of GFP-LC3-II-positive autophagic vesicles in H1299 cells. Accordingly, cell death rates 48 h after PICT-1 plasmid transfection were also significantly increased by PICT-1 overexpression (Figure 8G). In addition, confocal microscopy showed that PICT-1 overexpression did not obviously affect nucleolar structure or integrity of H H1299 (Figure 8H). These results further proved that PICT-1-induced autophagy is a pro-death form of autophagy, which does not involve nucleolar disruption and p53 activation.

## DISCUSSION

The nucleolus is the ribosome factory of the cell, where 47S precursor ribosomal RNAs are transcribed, cleaved, processed, and assembled with ribosomal proteins and 5S RNA to form ribosomal subunits [27,28]. In fact, ribosome biogenesis is a time- and space-regulated process tightly controlled by the cell cycle and the cellular microenvironment. Many nucleolar proteins have been found to participate in the regulation of rDNA transcription, pre-rRNA processing, and the assembly or transportation of ribosomal subunits. Dysregulation of ribosome biogenesis caused by the mutation of nucleolar regulatory proteins is associated with several human diseases, such as tumor genesis and progression [47–49]. PICT-1 has frequently been shown to be deleted or altered in human tumors, especially in gliomas [3,4]. Previous research showed that overexpression of PICT-1 inhibited proliferation and enhanced cell death of glioma cells by its direct interaction with and stabilization of PTEN. More recent work suggested that PICT-1 might play an important role in the regulation of ribosome biogenesis. In the present study, a ChIP assay showed that PICT-1 binds to rDNA genetic loci and that its overexpression inhibits the activation of UBF1 and the recruitment of Pol I to rDNA (Figure 4), thereby downregulating the transcription of the rDNA gene (Figure 3). Further, the 181-346 and 1-346 mutants that totally or partly lack nucleolus localization displayed markedly weaker inhibitory effects on the phosphorylation of UBF1 at ser388 and the transcription of the rDNA gene compared with full-length PICT-1 or the nucleolus-localized 181-479 mutant, suggesting that the inhibitory effects of PICT-1 are related to its nucleolar localization (Figure 2, 3 and 4). However, the particular protein region of PICT-1 that is responsible for its nucleolar functions still need to be identified in the future study.

In addition to its function as a ribosome factory, the nucleolus has been linked with numerous cellular activities including cell cycle control, development, viral replication, stress responses, senescence, cell death, and tumerogenesis [49]. While several recent studies have shown that the nucleolus also plays a role in autophagy [23,24], this relationship and the molecular mechanisms involved have not been fully characterized. In the present study, we found that PICT-1 overexpression markedly induced the formation of autophagic vesicles and the conversion of LC3-I to LC3-II in both U251 and MCF7 cells, suggesting that PICT-1 overexpression indeed caused autophagy in cancer cells (Figure 1). Nevertheless, the 181-346 and 1-346 mutant PICT-1 proteins, which totally or partially lack nucleolar localization and have a weaker inhibitory effect on rDNA transcription, exhibited significantly reduced abilities to induce autophagy in U251 cells, indicating that the blockade of rRNA transcription and ribosome biogenesis might be important causal factors in PICT-1-induced autophagy (Figure 2, 3 and 4). This hypothesis was further supported by the finding that CX5461, a specific inhibitor of Pol I polymerase, also significantly increased the induction of autophagy in U251 cells compared with untreated cells in a dose- and time-dependent manner (Figure 6). All of these results suggested that blocking ribosome biogenesis can induce autophagy in cancer cells. In fact, the relationship between inhibition of rDNA transcription and autophagy has been preliminarily investigated by several researchers. They have also found that treatment with Pol I inhibitors such as adriamycin (ADR) and actinomycin D (Act D), or with specific siRNAs for the Pol I transcription co-factor TIF-IA and the Pol I catalytic subunit POLR1A, resulted in significant inhibition of Pol-mediated transcription and autophagy [24,50–52]. However, we hypothesized that PICT-1-induced autophagy might be different from autophagy induced by these rRNA transcriptional inhibitors, because disruption of the nucleolar structure is observed in inhibitor-treated cells [24,50–52]. Recent studies have shown that nucleolar disruption leads to translocation of many nucleolar proteins from the nucleolus to the nucleoplasm. These proteins include nucleophosmin, RPS7, RPL5, RPL11, and RPL23 [53], which can cause the accumulation and activation of p53 by associating with MDM2, thus inhibiting MDM2-mediated ubiquitination and degradation of p53. More importantly, p53 activation has been implicated in nucleolar stress-induced autophagy [23,53]. Therefore, nucleolar disruption-induced autophagy might be a mixed result. One possible cause is the inhibition of ribosomal biogenesis, while another is the translocation of nucleolar proteins; however, it is difficult to distinguish their roles in this process. In the present study, we found that PICT-1 overexpression did not obviously affect the integrity of nucleolar structure and p53 expression levels (Figure 8E and 8H), indicating that the dysregulation of ribosomal biogenesis is the major cause of PICT-1-induced autophagy. In fact, It has been reported that PICT-1 can directly bind 5S rRNA and is required for the integration of 5S RNP into the ribosome [54]. Thus, our results together with previous report indicated PICT-1 is an essential regulator of ribosome biogenesis, which regulates ribosome biogenesis not only at the stage of pre-rRNA biosynthesis, but also at the stage of ribosomal subunits assembly.

The mTOR/p70S6K signaling pathway plays a key role in the regulation of not only cell survival and proliferation, but also autophagy [18,45]. In the present study we demonstrated that both PICT-1 overexpression and CX-5461 treatment inhibit the AKT/mTOR/p70S6K transduction pathway, indicating that autophagy induced by the inhibition of rRNA transcription also regulates the AKT/mTOR/p70S6K pathway. However, the mechanism by which AKT/mTOR/p70S6K pathway activity is modulated by the dysregulation of ribosome biogenesis is still not entirely clear.

**Figure 9:**
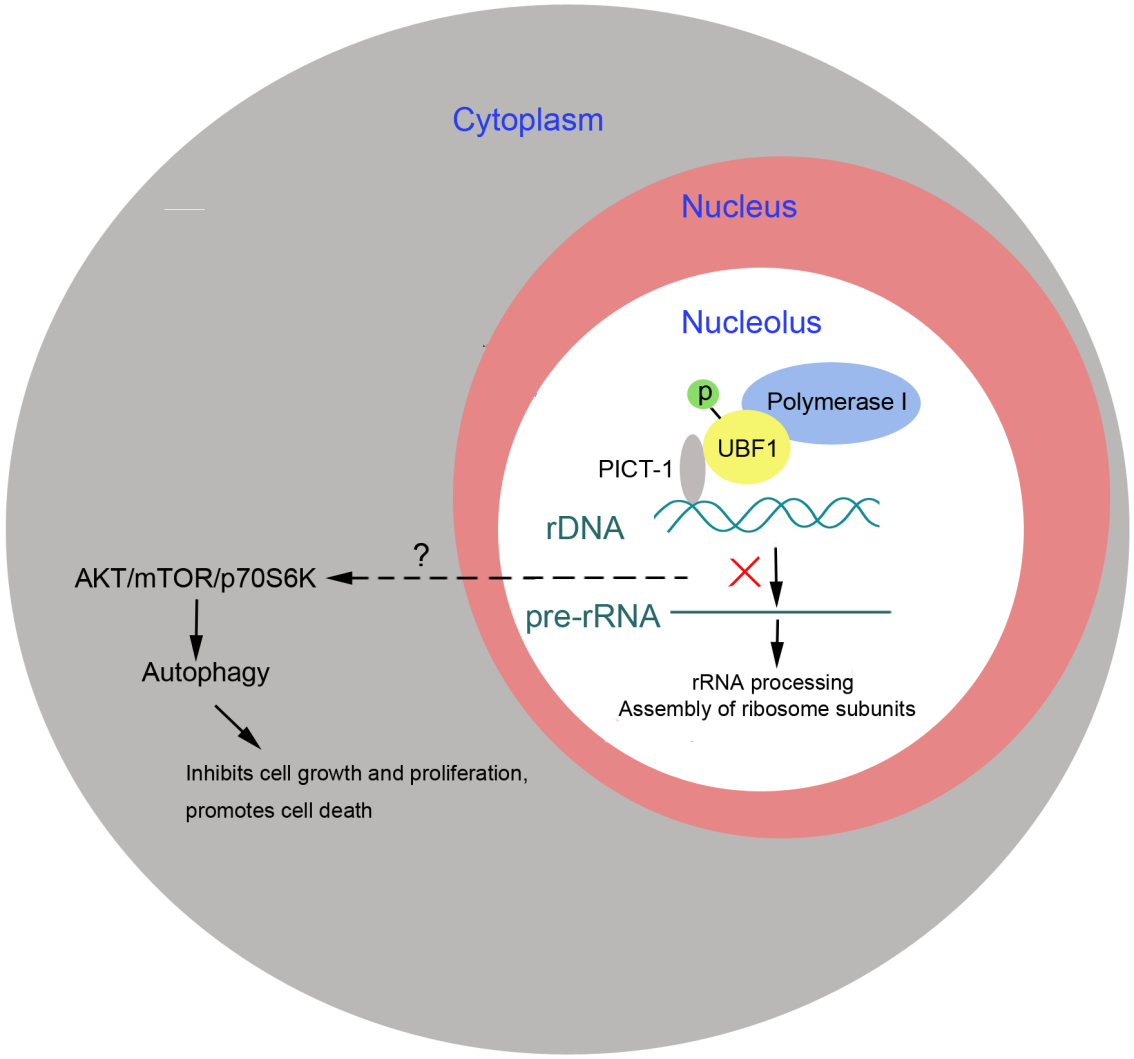
PICT-1 functions as a tumor suppressor to induce autophagy-related cell growth inhibition and cell death PICT-1 overexpression induces autophagy-related inhibition of cell growth and cell death, which is implicated in the nucleolar localization of PICT-1, inhibition of rRNA transcription, and inactivation of the ATK/Mtor/p70S6K signal pathway.

Depending on the particular cell types and cellular stresses involved, autophagy seems to have different roles, either promoting cancer cell survival or triggering cell death. In our investigation, 3-MA treatment markedly enhanced the rate of cell death induced by PICT-1 overexpression, suggesting that PICT-1-induced autophagy is a pro-death rather than a pro-survival process. Thus, our results reveal that besides its PTEN-dependent anti-cancer effects, PICT-1 can also exhibit tumor suppressor function by triggering a pro-death autophagic process which is involves rRNA transcription inhibition and the inactivation of the AKT/mTOR/p70S6K transduction pathway (Figure 9).

## MATERIALS AND METHODS

### Cell lines and reagents

Human U251 glioblastoma and human MCF7 breast adenocarcinoma cell lines were purchased from the American Type Culture Collection (ATCC). The cells were cultured in DMEM medium containing 10% foetal bovine serum (FBS) and antibiotics (100 IU/mL penicillin and 100 μg/mL streptomycin). CX-5461 was purchased from Selleck Chemicals (Houston, TX, USA). The primary antibodies for p-UBF1 (ser 388), UBF1, RPA194, nucleolin, PICT-1, p53, and ß-actin were purchased from Santa Cruz Biotechnology (Santa Cruz, CA, USA). Primary antibodies against AKT, p-AKT (S473), p-mTOR (S2448), mTOR, p-p70S6K (S371), and LC3 were obtained from Cell Signaling (Beverly, MA, USA). Anti-FLAG antibody was purchased from Sigma–Aldrich (St. Louis, MO, USA). HRP- and fluorescein-labeled secondary antibodies and the ECL kit were purchased from KPL (Gaithersburg, MD, USA). The pGFPc1-LC3 plasmid was a gift from Marja Jäättelä. The pHrD-IRES-Luc plasmid containing the rDNA promoter sequence was a kind gift of professors Ke Y and Jacob ST [33,34]. PICT-1 and its deletion mutants (aa 1–346, aa 181–346, aa 181–479) were cloned into pEGFPc1 (Clontech, Palo Alto, CA, USA), pDsRedc1 (Clontech), or p-FLAG-CMV2 (Sigma–Aldrich) to generate green fluorescent protein (GFP)-tagged, red fluorescent protein (RFP)-tagged, or FLAG-tagged fusion proteins. Lipofectamine 2000 was purchased from Invitrogen (Grand Island, NY, USA). 4',6-Diamidino-2-phenylindole (DAPI), actinomycin D (Act D), bafilomycin A1 (BAF) and 3-methyl-adenine (3-MA) were purchased from Sigma Chemical Co. (St. Louis, MO, USA). Co-immunoprecipitation (Co-IP) assays were performed using protein G Sepharose beads (GE Healthcare, Piscataway, NJ), and chromatin immunoprecipitation (ChIP) assays were performed using Dynabeads Protein G magnetic beads (Life Technologies, Carlsbad, CA, USA). Luciferase assays were performed using a luciferase reporter assay kit (Promega, Madison, WI, USA). Total RNA was isolated with Trizol reagent (Invitrogen) and real-time quantitative PCR (qPCR) was performed using a SYBR Green real-time PCR kit (Toyobo, Osaka, Japan).

### Cell proliferation assay

Cells were seeded at a density of 0.2 × 10^6^ cells/10 cm plate and transfected with the vectors pFLAG-CMV2, pFLAG-CMV2-PICT-1, pFLAG-CMV2-PICT-1 (1-346), pFLAG-CMV2-PICT-1 (181-346), or pFLAG-CMV2-PICT-1 (181-479), respectively. Cell numbers were calculated every day and data from three independent experiments were presented as mean ± standard deviation (SD).

### Western blotting

Cells were harvested and lysed in cell lysis buffer (10 mM EDTA, 1% SDS, 50 mM Tris-HCl (pH 8.0) and 0.1 mM PMSF). Equal amounts of protein were separated by SDS-PAGE and transferred onto PVDF membranes. The membranes were blocked with 5% non-fat milk in TBS-T (0.1% TWEEN 20) for 2 h at room temperature (RT) and then probed with the indicated primary antibodies overnight at 4°C. The specific bands were detected using ECL reagents after a second incubation with appropriate secondary antibodies.

### Quantitative real-time PCR (qPCR) for pre-rRNA and c-myc

Cells were transfected with pFLAG-CMV2 vector, pFLAG-CMV2-PICT-1, pFLAG-CMV2-PICT-1 (1-346), pFLAG-CMV2-PICT-1 (181-346), or pFLAG-CMV2-PICT-1 (181-479) for 48 h, respectively. Total RNA was extracted using TRIzol regent and cDNA was synthesized using random primers. The cDNA products were used to evaluate pre-rRNA expression levels using SYBR Green I on an ABI7300 machine. Primers for pre-rRNA and β-actin used for qPCR were synthesized as described in previous reports [31,32]. Primers for c-myc were as follows: Forward, 5′-AATGAAAAGGCCCCCAAGGTAGTTATCC-3′; Reverse: 5′-GTCGTTTCCGCAACAAGTCCTCTTC-3′.

### Luciferase reporter assay

Cells were co-transfected with pHrD-IRES-Luc and pFLAG-CMV2-PICT-1 or its mutant plasmids for 48 h, harvested, and lysed in passive lysis buffer. Cell lysates containing same amount of protein were subjected to luciferase activity analysis using a luciferase assay kit (Promega).

### Fluorescence detection by confocal microscopy

For autophagy detection, cells grown on sterile coverslips were transfected with pGFP-LC3, pDsRed-PICT-1, or its mutant plasmids together with or without CX-5461 treatment were fixed for 30 min in 4% formaldehyde, and then permeabilized in PBS containing 0.1% Triton X100 for another 30 min at RT. After staining with 0.5 μg/mL of DAPI for 10 min, fluorescence of GFP, RFP, or DAPI was observed using an Olympus FV1000 confocal microscope. The number of GFP-LC3 puncta per cell was quantified. More than 30 cells were analyzed in each condition and the results are the means of three independent experiments.

For the investigation of PICT-1 sub-nucleolar localization, after fixation and permeabilization were performed as described above, cells were blocked with 1% BSA for 1 h at RT and probed with the appropriate primary antibody at 4°C overnight. The coverslips were incubated with rhodamine- and fluorescein-conjugated secondary antibodies for 1 h at RT. After staining with DAPI, the cells were visualized and imaged on an Olympus FV1000 confocal microscope.

### Co-immunoprecipitation assay (Co-IP)

Cells were transfected with pFLAG-CMV2-PICT-1 for 24 h, then lysed in IP buffer (1 mM EDTA, 1 mM EGTA, 150 mM NaCl, 1% Triton X-100, protease inhibitor cocktail, 20 mM Tris-HCl pH 8.0) with sonication for 10 sec. Cell lysates were then incubated with protein G agarose and control IgG or the indicated primary antibodies for 4 h at RT. The immunoprecipited proteins were subjected to Western blotting using the indicated antibodies.

### Chromatin immunoprecipitation (ChIP)

ChIPs were performed as described in a previous report with minor modifications [55]. Briefly, U251 cells were fixed with 1% formaldehyde for 15 min at RT, and then quenched with 125 mM glycine for 5 min. The cells were washed three times with PBS and lysed with a lysis buffer (5 mM EDTA, 0.5% NP-40, 150 mM NaCl, 50 mM Tris-HCl pH 7.5, 1.0% Triton-X-100, and protease inhibitor cocktail). Cell lysates containing 200-1000bp DNA fragments were produced by sonication and incubated with the indicated antibodies or control IgG together with Dynabeads Protein G magnetic beads for 4 h at RT. After washing three times with lysis buffer, beads were resuspended with Chelex 100 suspension with proteinase K for 30 min at RT, boiled for 10 min, and cooled on ice. After shaking (55°C at 1,400 rpm) for 1 h, the beads were boiled for another 10 min and suspension was collected by centrifugation to perform qPCR analysis using the previous reported primers (H0, H1, H4, H8, H13, H18, H23, H42.9) [36].

### Cell death analysis by DAPI staining

Cells were co-transfected with pGFP-LC3 and pFLAG-CMV2 or pFLAG-CMV2-PICT-1 in the presence or absence of 3-MA for 48 h. Cells were fixed in pre-chilled methanol for 5 min, stained with 0.5 μg/mL of DAPI for 10 min, and visualized on an Olympus FV1000 confocal microscope. Dead cells with fragmented or condensed nuclei were counted and used to calculate cell death rates.

### Statistical analysis

Data taken from three independent experiments were expressed as mean ± standard deviation (SD). Student's *t*-test was used to compare the variables in this study. Significance was set at a value of *p* < 0.05.
